# A Dual-Crosslinked and Anisotropic Regenerated Cellulose/Boron Nitride Nanosheets Film With High Thermal Conductivity, Mechanical Strength, and Toughness

**DOI:** 10.3389/fbioe.2020.602318

**Published:** 2020-12-18

**Authors:** Xuran Xu, Yichuan Su, Yongzheng Zhang, Shuaining Wu, Kai Wu, Qiang Fu

**Affiliations:** ^1^State Key Laboratory of Polymer Materials Engineering, College of Polymer Science and Engineering, Sichuan University, Chengdu, China; ^2^Key Laboratory for Soft Chemistry and Functional Materials of the Ministry of Education, School of Chemical Engineering, Nanjing University of Science and Technology, Nanjing, China; ^3^Key Laboratory of Advanced Technologies of Materials, Ministry of Education China, Southwest Jiaotong University, Chengdu, China

**Keywords:** regenerated cellulose, thermal conductivity, boron nitride nanosheets, dual-crosslink, urea

## Abstract

The highly thermo-conductive but electrically insulating film, with desirable mechanical performances, is extremely demanded for thermal management of portable and wearable electronics. The integration of boron nitride nanosheets (BNNSs) with regenerated cellulose (RC) is a sustainable strategy to satisfy these requirements, while its practical application is still restricted by the brittle fracture and loss of toughness of the composite films especially at the high BNNS addition. Herein, a dual-crosslinked strategy accompanied with uniaxial pre-stretching treatment was introduced to engineer the artificial RC/BNNS film, in which partial chemical bonding interactions enable the effective interfiber slippage and prevent any mechanical fracture, while non-covalent hydrogen bonding interactions serve as the sacrifice bonds to dissipate the stress energy, resulting in a simultaneous high mechanical strength (103.4 MPa) and toughness (10.2 MJ/m^3^) at the BNNS content of 45 wt%. More importantly, attributed to the highly anisotropic configuration of BNNS, the RC/BNNS composite film also behaves as an extraordinary in-plane thermal conductivity of 15.2 W/m·K. Along with additional favorable water resistance and bending tolerance, this tactfully engineered film ensures promised applications for heat dissipation in powerful electronic devices.

## Introduction

With the rapid development of the highly integrated electronic devices toward high power and increasing miniaturization, research on the flexible thermo-conductive material becomes urgent to satisfy the thermal management requirements in portable and wearable applications (Li et al., 2015; Chen et al., 2018a; Wu et al., 2020). Regenerated cellulose (RC), a green and biodegradable biomass macromolecule that is constructed simply by physical dissolution and then regeneration into the fibrous configuration without abundant consumption of chemical reagents (Ray et al., 2020; Tu et al., 2020; Wågberg and Erlandsson, 2020), is a sustainable choice for conformation of thermo-conductive paper-like material, broadening its extensive usage in our real life (Wang et al., 2016; Zhu et al., 2016). Typically, boron nitride nanosheets (BNNS), one type of two-dimensional thermo-conductive but electrically insulating nanofiller (Weng et al., 2016), with the prominent basal thermal conductivity (TC) up to 400 W/m·K (Guerra et al., 2019), is attractive for integration with RC for the fabrication of a thermo-conductive film (Yao et al., 2016; Ye et al., 2019). The essential issue is to balance the contradiction between tremendous enhancement of TC, and simultaneous achievement of high mechanical strength and toughness, which remains a great challenge (Yao et al., 2018; Yin et al., 2019).

In the past, some researches have tried to hybrid BNNS with RC for fabrication of flexible RC/BNNS nanocomposite films (Pickering et al., 2016; Wu K. et al., 2019; Li et al., 2020). For example, a green pathway that is using NaOH/urea aqueous solution as the dissolving agent was reported (Cai and Zhang, 2005), in which BNNSs were intensely mixed with RC by direct mechanical stirring, resulting in an in-plane TC of 2.97 W/m K, mechanical strength of 70 MPa, and elongation of 14% at a BNNS addition of 30 wt% (Lao et al., 2018). These limited in-plane TC, mechanical strength, and toughness are mainly attributed to the following reasons: (i) Low BNNS addition, derived from the challenging dispersion of BNNSs in aqueous solution of cellulose, leads to insufficient BNNS–BNNS interconnections and thus discontinuous phonon pathways within the RC matrix (Cai et al., 2017; Ma et al., 2020). (ii) Isotropic alignment of BNNS without adequate in-plane orientation could not take advantage of the highly thermo-conductive trait of BNNSs to the fullest (Wang et al., 2019; Liu et al., 2020). (iii) Weak bonding interactions merely by intermolecular hydrogen bonds of cellulose and their random molecular configuration is easy to give rise to mechanical fracture during uniaxial stretching, without enough interfiber slippage to dissipate the stress energy (Osorio-Madrazo et al., 2012; Tu et al., 2020).

The key to achieve simultaneous high mechanical properties and in-plane TC is to tactfully tailor the structural features of building blocks (BNNS and cellulose nanofiber) with favorable arrangements in the in-plane direction (Yuan et al., 2015; Cho et al., 2016; Han et al., 2019), resulting in the formation of highly anisotropic configurations (Zhang et al., 2018; Tarhini and Tehranibagha, 2019). Moreover, in comparison with self-assembled fibrous nanocellulose, the RC film built from regeneration and then assembly of cellulose chains is provided with much more tunable chemistry and functionality (Li et al., 2018; Wu Y. et al., 2019; Yang et al., 2019). For example, a synergistic strategy based on chemical (interfiber covalent crosslinking) and physical (non-covalent interactions via the hydrogen bonds) crosslinks is effective to tailor the structural characteristics for the purpose of high mechanical properties (Rodell et al., 2016; Song et al., 2017; Liu et al., 2018).

In this study, a dual-crosslinking assisted with pre-orientation strategy is adopted to engineer the thermo-conductive RC/BNNS composite film, in which epoxy chloropropane (ECH) was used for the covalent crosslinking, while the RC nanofibers are also non-covalently bonded by hydrogen bonding interaction between adjacent RC nanofibers. Compared with the conventional vacuum–filtration strategy (Yang et al., 2017; Cao et al., 2018; Chen et al., 2018b) in which nanocellulose is mainly assembled merely via intermolecular hydrogen interactions, this synergistic effect not only enables the effective interfiber slippage and prevent any mechanical fracture through partial chemical bonding interactions but also non-covalent hydrogen bonding interactions serve as the sacrifice bonds to dissipate the stress energy, ensuring a simultaneous high mechanical strength and toughness at the high loading of BNNSs (45 wt%). More importantly, ascribed to the high-loading but homogeneous BNNS, and the anisotropic BNNS alignment due to the uniaxial stretching treatment, the RC/BNNS composite film also behaves at an extraordinary in-plane thermal conductivity up to 15.2 W/m·K. Along with an additional favorable water resistance and bending tolerance (>10,000 times), this tactfully engineered film broadens its applications in next-generation portable and wearable electronic devices for efficient heat dissipation.

## Materials and Methods

### Materials

Lithium hydroxide (LiOH, 98%), lithium chloride (LiCl, 99.0%), phytic acid (70%), epoxy chloropropane (ECH, 99.5%), and urea (99.0%) were obtained from Aladdin Reagent Co., Ltd. Cotton linter pulp was obtained from Jilin Chemical Fiber Group Co., Ltd. These chemicals were used as received without further purification. Boron nitride (99.9%, 12 μm) was obtained from Sigma-Aldrich Co., Ltd and was exfoliated according to our previous study.

### Fabrication of the Dual-Crosslinked RC/BNNS Films

Dual-crosslinked RC/BNNS composite films were fabricated as shown in Figure S1. First, cotton linter pulp was immersed in a precooled 4.6 wt% urea/15 wt% LiOH aqueous solvent (−12°C) and stirred at 950 rpm until the temperature approached to 5°C, then the solution in liquid nitrogen was quickly frozen and diluted to 6 wt%. The stirring and freezing procedures were repeated until the solution was clear and sticky. Simultaneously, the BNNSs were immersed in the above LiOH/urea solvent under stirring at 500 rpm, following a ball milling process for 2 h, and then frozen in liquid nitrogen. Afterward, 21.2 g of the cellulose solution was mixed with the BNNS solution at different mass ratios of BNNS/cellulose from 0 to 60 wt%, and 0.23 g ECH was added into the mixture under stirring at 950 rpm. After defoaming, the solution was poured into molds and kept at 5°C for 30 h. Subsequently, the as-prepared RC/BNNS gels were pre-stretched to specific strains ranging from 100 to 400% and then immersed in the 5-wt% phytic acid aqueous solution for 30 min to achieve the physical crosslinking. Last, the gels were immersed in deionized water for 48 h, and the anisotropic and dual-crosslinked RC/BNNS films were obtained by air drying the gels at room temperature. To verify the effect of RC fibers on the structure and mechanical properties of the materials, a merely physical cross-linked RC film and dual-crosslinked RC films with 100 and 300% pre-stretching were also prepared without RNNS addition for comparison.

### Characterization

The structures and morphologies of the fabricated samples were observed by atomic force microscopy (AFM, Bruker Veeco Multimode 8), scanning electron microscopy (SEM, Hitachi S-4800), and transmission electron microscopy (TEM, TECNAI G2-20 LaB6). The Fourier transform infrared (FT-IR) spectra were collected on a Thermo Scientific Nicolet iS5 spectrometer with scanning wavelengths from 500 to 4,000 cm^−1^. The 2D wide-angle X-ray diffraction (WAXD) measurements were performed on a WAXD diffractometer (Rigaku D/MAX-1200). The X-ray diffraction (XRD) patterns were obtained by using a Bruker AXS D8 advanced diffractometer. The dispersion degree of the suspension was analyzed by a polarizing microscope (BM2100 POL). The TC of the sample was calculated according the equation TC = α × ρ × C, where α, ρ, and C, correspond to the thermal diffusivity, density, and specific heat capacity of the sample, respectively. The in-plane and out-of-plane thermal diffusivity of the samples were measured by a flash thermal conductivity analyzer (Netzsch LFA 467) at a voltage of 250 V and pulse width of 300 μs. The rheological properties of the samples were tested by rotational rheometer (TA AR2000ex). Thermogravimetric analysis was carried out on a synchronized thermogravimetric analyzer (TGA/DSC, Mettler Toledo TGA/DSC1/1100LF), and the samples were heated from 25 to 700°C at a heating rate of 20°C/min. Surface wettability of the samples were tested by a contact angle tester (Solon SL200B).

### Finite Element Simulation

Finite element analysis was carried out to visualize the temperature and heat flux distribution within RCF/BNNS during heat dissipation. The simulation was based on classical Fourier Law [q = –κ × grad (T)], where q, κ, and grad (T) corresponds to heat flux, thermal conductivity, and temperature gradient, respectively.

## Results and Discussions

**Figure 1:**
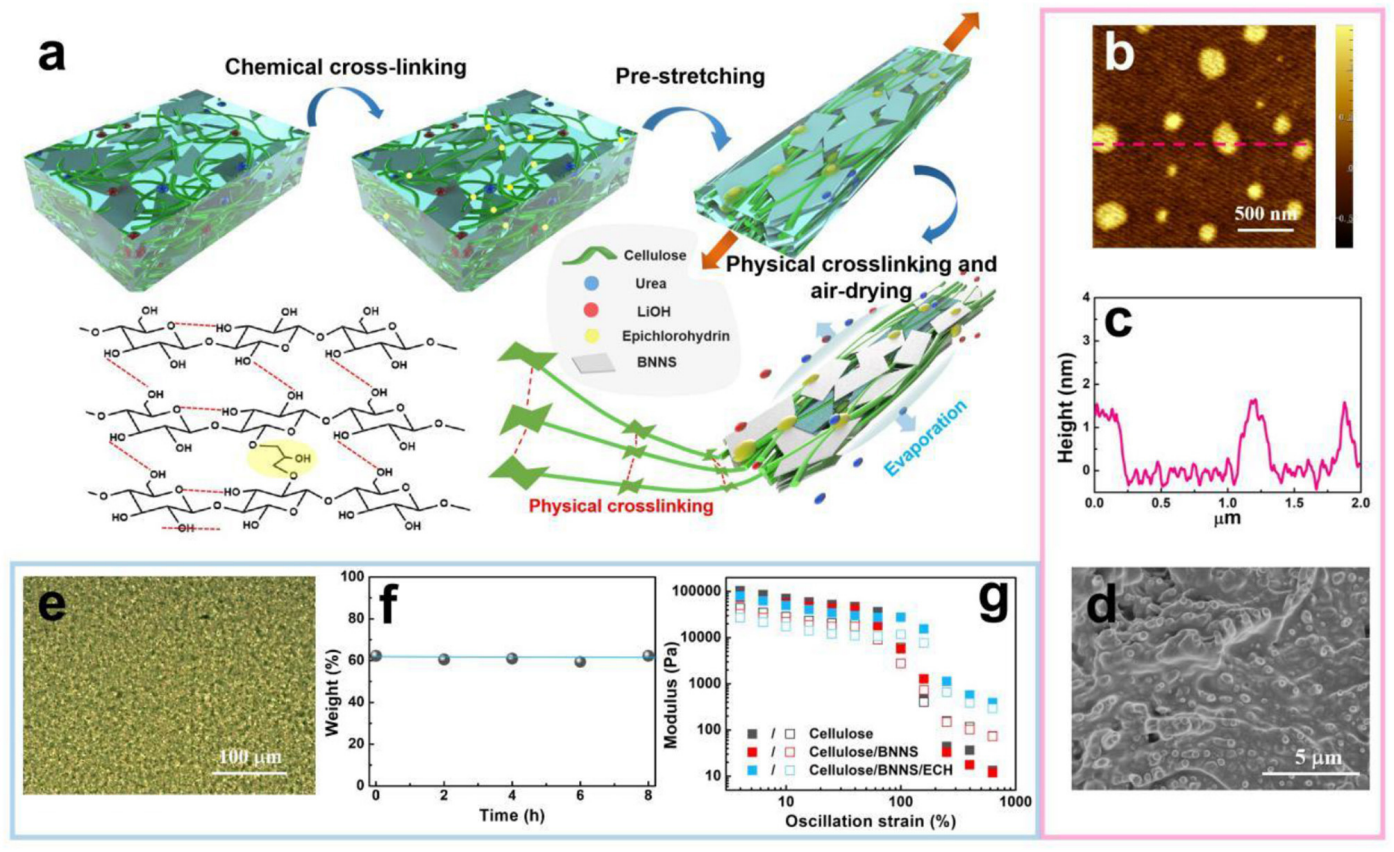
**(a)** Schematic illustration of the fabrication process of the dual-crosslinked regenerated cellulose (RC)/boron nitride nanosheet (BNNS) film. **(b)** Atomic force microscopy (AFM) micrograph and **(c)** the corresponding height information of BNNS. **(d)** *In situ* scanning electron microscopy (SEM) image of cellulose nanofibers within the precipitated RC paper. **(e)** Microscopic image of the cellulose/BNNS suspension. **(f)** Weight fraction of BNNS in the supernatant of cellulose/BNNS suspension by thermogravimetric analyzer (TGA) test. **(g)** Rheological test results of the cellulose, cellulose/BNNS, and cellulose/BNNS/epoxy chloropropane (ECH) suspension.

The anisotropic and dual-crosslinked RC/BNNS films were well-designed through a pre-orientation assisted with dual cross-linking strategy (Figure 1a). First of all, so as to improve the compatibility of RC and BNNS, BNNSs with a lateral size of 200–300 nm and thickness of 1–2 nm (Figures 1b,c) were fabricated with a typical ball-milling treatment with alkali/urea solvent. Dissolved cellulose solution was obtained via a typical alkali/urea solvent, in which abundant cellulose nanofibers with a diameter of ~30 nm exist in the solution (Figure 1d). After mixing them together, the cellulose/BNNS suspension could present a very uniform dispersion according to the optical microscope result (Figure 1e, no obvious BNNS aggregations), and it was verified that the BNNS sheets could remain stable and dispersive in the suspension after long standing according to the TGA test of the supernatant (Figure 1f and Figure S2). Epichlorohydrin (ECH) was employed to give rise to firm chemical crosslinked bonds by introducing the epoxy groups, which would crosslink the adjacent RC nanofibers, leading to the construction of the chemical gels with a sudden increase in viscosity (Figure S3). The rheological test results (Figure 1g) show that, as the oscillation strain increased, both the cellulose solution and the cellulose/BNNS suspension possessed higher values of loss modulus than that of the storage modulus, behaving in a liquid-like state (Zhang et al., 2020), while the RC/BNNS/ECH composite exhibited the opposite result, showing a gel-like state, which further proved the formation of the hydroxyl gel. However, the cross-linking amount of such homogeneous and isotropic gel was low, and its mechanical strength is needed to be further improved for practical application. Therefore, a stretch force was applied to both sides of the gel to guide the anisotropic arrangement of the RC chains and BNNS, following a phytic acid bath to destroy the alkali/urea solvent shells and fix the pre-stretched structure of the composites. Upon pre-stretching, the RC chain exhibited an anisotropic arrangement along the direction of the stretching force. Simultaneously, the stretch force of pre-stretching and restriction of RC endow the BNNS with an orientated topology in the film. In the wake of phytic acid bath, the alkali/urea solvent system, which was broken and physical crosslinked within the hydrogen bond, was therefore formed. Finally, the dual cross-linked RC/BNNS hydrogels were fixed to prevent the shrinkage and air dried to form the anisotropic structure of the RC and BNNS, which is expected to achieve a much higher tensile strength and in-plane thermal conductivity of the films.

**Figure 2:**
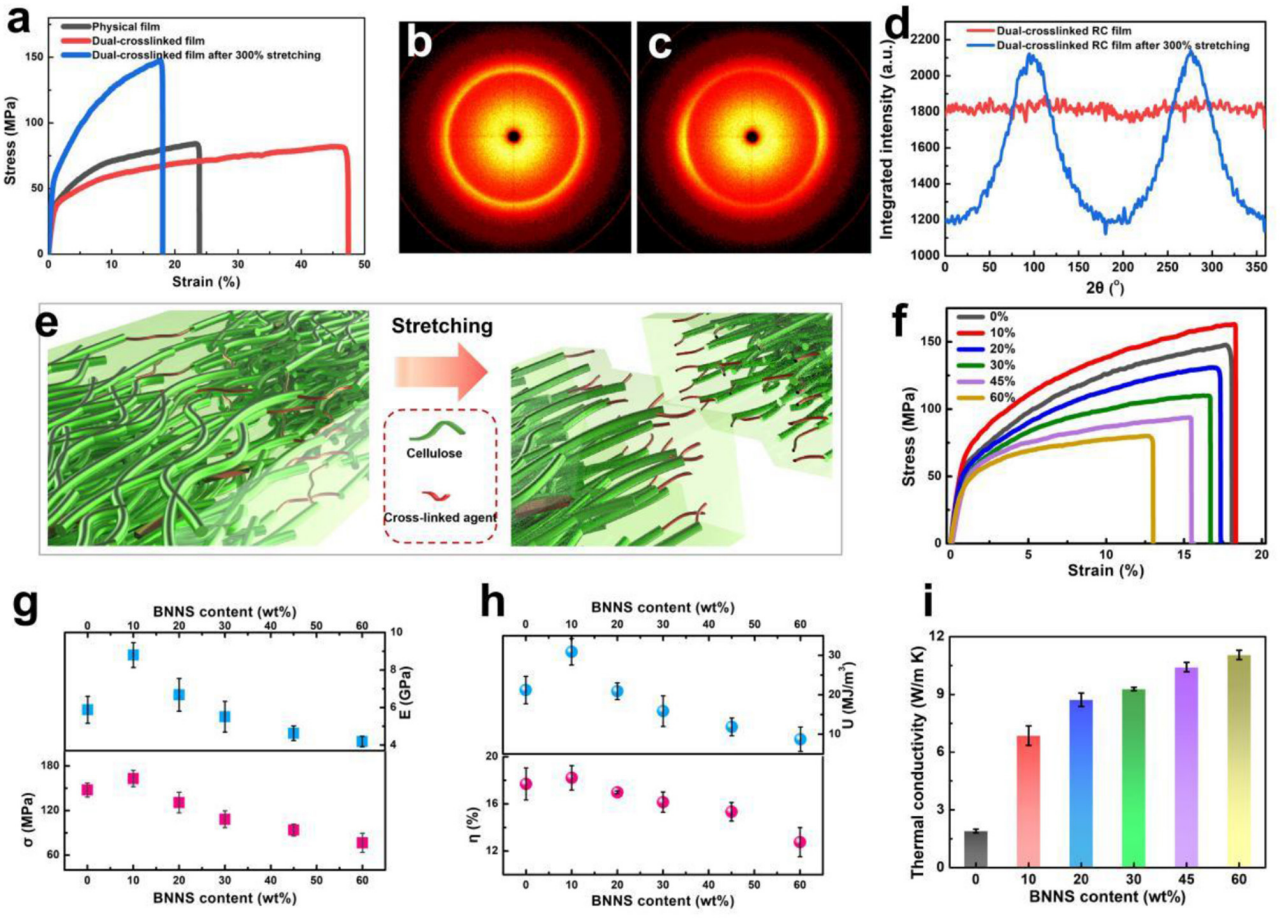
**(a)** Stress–strain curves of the RC films with different crosslinking states; WAXS patterns of **(b)** the dual-crosslinked RC film without pre-stretching and **(c)** the dual-crosslinked RC film after 300% stretching, and **(d)** the corresponding azimuthal-integrated intensity distribution curves. **(e)** Mechanism diagram of the dual-crosslinking enhancement of the RC/BNNS film. **(f)** Stress–strain curves, **(g)** modulus and tensile strength, **(h)** toughness and elongation at break, and **(i)** thermal conductivities of the dual-crosslinked RC/BNNS film with 0–60 wt% of BNNS additions.

The FT-IR spectra of the physical crosslinked RC, dual-crosslinked RC, physical crosslinked RC/BNNS, and dual-crosslinked RC/BNNS films confirmed the reaction and hydrogen bond interactions between the cellulose chains of the samples (Figure S4). The physical crosslinked RC film exhibited a series of characteristic peaks at 3,450, 2,880, and 1,644 cm^−1^ that correspond to –OH stretching, –CH_2_ stretching, and H–O–H stretching, respectively. The absorption bands of the RC were 1,350, 1,160, and 1,060 cm^−1^, which were attributed to C–H scissor vibration, –OH wagging vibration, and C–O and C–C stretching vibration, respectively (Gao et al., 2011; Zhao et al., 2016). However, after crosslinking by the ECH, the intensities of these absorption peaks decreased. This result indicated that the hydroxyl groups on the cellulose chains partially reacted with ECH to form covalent crosslinking. After blending with BNNS nanosheets, two typical peaks of BN at 819 cm^−1^ (deformation of B–N bonds) and 1,370 cm^−1^ (stretching vibrations of B–N bonds) appeared in the spectra of both physical crosslinked and dual-crosslinked RC/BNNS films (Yu et al., 2016). The thermal degradation behavior of RC and RC/BNNS films with and without chemical crosslinking were compared by TGA and DSC curves (Figure S5). The physical crosslinked RC film exhibited a major weight loss between 300 and 400°C, while the initial decomposition temperature of the dual-crosslinked RC film was relatively lower, and the residual weight ratio was a little higher than the physical crosslinked RC film due to the existence of the ECH. After adding the BNNS, the residual weight ratios of both of the films were increased remarkably, which were consistent with the mass ratios of BNNSs. To clarify the impact of the dual-crosslinked structure and stretching effect on the material properties, the stress–strain curves of the pristine RC, dual-crosslinked RC, and dual-crosslinked RC in 300% stretch film were compared as shown in Figure 2a. For the RC film with only physical crosslinking by hydrogen bonding interactions, the tensile strength and elongation at break were 84.2 MPa and 23.1%, respectively. Nevertheless, the introduction of the chemical cross-linking effect of ECH was used as a kind of supporting bond to prevent the interfiber slippage in the stretching process. The elongation at break of the film increased to 45.7%, while the tensile strength was almost unchanged, which was attributed to the slip of the RC chain before break and chemical crosslink between the RC nanofiber. Furthermore, when the RC film was pre-stretched to a strain of 300%, the cellulose molecular chains or embedded cellulose nanofibers would be induced to orientation, and the slip distance between molecular chains or nanofibers would be reduced, which not only greatly improved the tensile strength of the film but also maintained a high material toughness under the interaction of dual-crosslinking. Figures 2b–d show the WAXS patterns and their corresponding orientation factors of the dual-crosslinked RC films after 100 and 300% stretching in a synchrotron radiation facility. For the film without stretching, the WAXS pattern shows a very uniform diffraction pattern at all azimuthal angles. Differently, the WAXS pattern of the dual-crosslinked RC film with 300% stretching shows clear equatorial arcs with two obvious peaks of integrated intensity, revealing the changes of the dual-crosslinked structure from isotropy to anisotropy (Pan et al., 2019; Wang et al., 2020). Therefore, as illustrated in Figure 2e, it is considered that the dual interactions could provide prominent mechanical enhancement, in which chemical crosslinking interactions offer the effective interfiber slip and prevent any fracture, while intermolecular hydrogen bonding interactions serve as the sacrifice bonds to dissipate the stress energy, resulting in a simultaneous high mechanical strength and toughness.

To further study the effect of BNNS content on the dual-crosslinked composite films, taking a stretching ratio of 300% as an example, the pre-stretched RC/BNNS samples with a BNNS weight ratio from 0 to 60 wt% were compared through stress–strain test in Figure 2f. Compared with pure RC film, when 10 wt% of BNNS was added, the tensile strength and elongation at break of the RC film was slightly improved to 163.1 MPa and 18.2%, respectively. It is due to the good interfacial interaction between RC and BNNS, which deliver the force from RC to BNNS when stretched. However, with the increase in BNNS content, more defects between excess BNNS and the substrate occurred, leading to gradual decrease in the tensile strength, elongation at break, toughness, and modulus of the composite film (Figures 2g,h). Especially, when the BNNS content increased from 45 to 60 wt%, the toughness and elongation at break of the composite film decreased significantly, which were caused by the structural defects as shown in Figure S6. Besides, the thermal conductivities of the above samples are compared in Figure 2i. It is noted that the in-plane-orientated BNNS obviously enhanced the thermal conductivity, indicating that the thermally conductive path was formed (Wu Y. et al., 2019). With the increase in BNNS content, abundant thermal conductive phonon pathways were established, and the thermal conductivity of the composite film was increased accordingly. When the BNNS content increased from 45 to 60 wt%, the thermal conductivity was increasing not obviously from 10.4 to 11.0 W/m·K, corresponding to the beforehand establishment of the conductive path. Therefore, the dual cross-linking strategy accompanied by the optimal BNNS content of 45 wt% was chosen to prepare the highly thermo-conductive RC/BNNS film.

**Figure 3:**
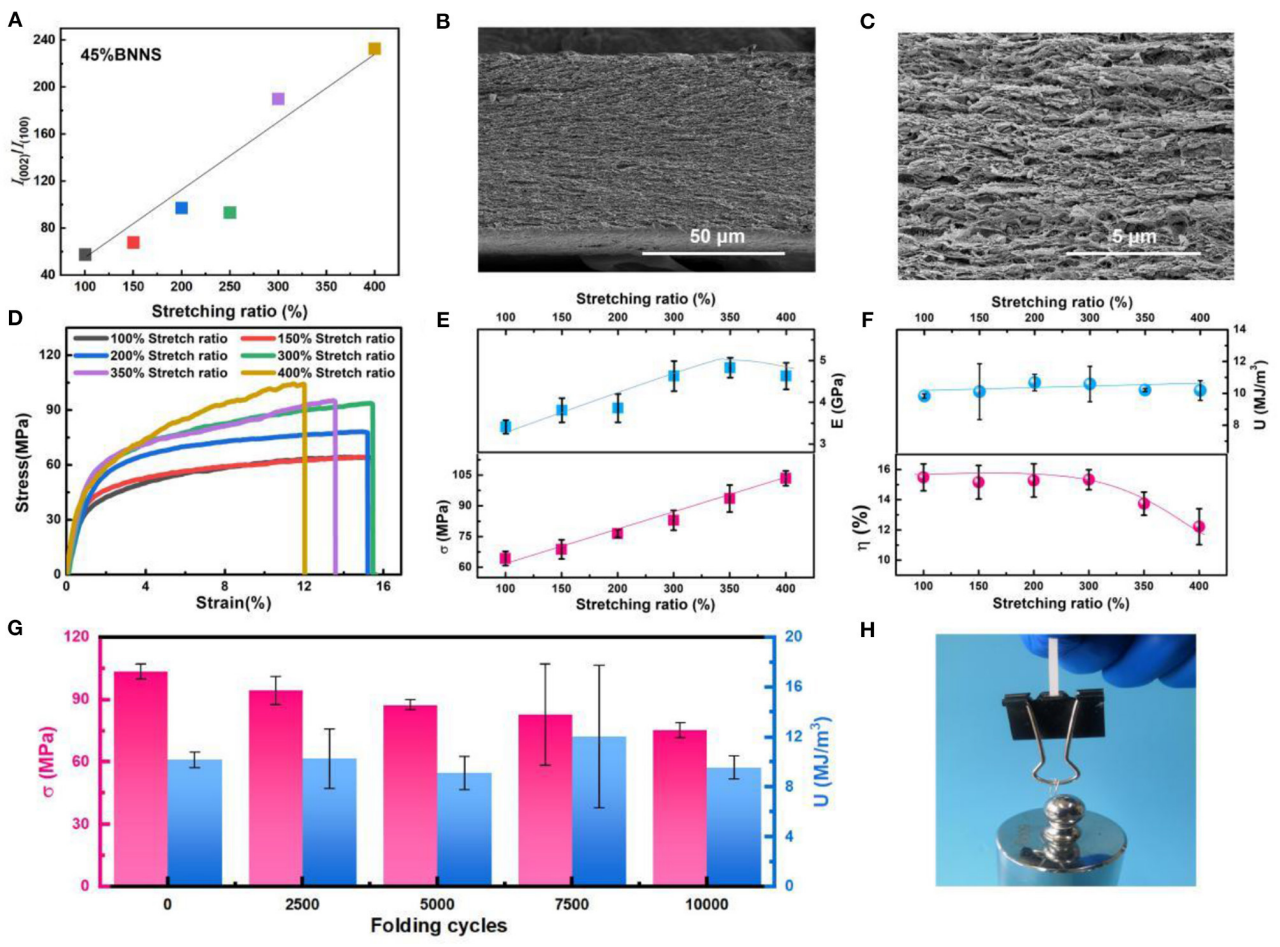
**(A)** *I*_002_*/I*_100_values calculated from the X-ray diffraction (XRD) information of the dual-crosslinked RC/BNNS films after 100–400% stretching. **(B)** Cross-section SEM image and **(C)** the partial enlarged detail of the dual-crosslinked RC/BNNS film after 400% stretching. **(D)** Stress–strain curves, **(E)** tensile strength and modulus, **(F)** elongation at break and toughness of the dual-crosslinked RC/BNNS films after 100–400% stretching. **(G)** Tensile strength and toughness after repeated folding cycles. **(H)** Digital image of the folded RC/BNNS film after 400% stretching that can withstand a weight of 500 g.

The structural alignment of the BNNS in the dual-crosslinked RC/BNNS film is mainly induced by the pre-stretching before the physical crosslinking process. During pre-stretching, the BNNSs were limited in an in-plane-oriented arrangement, which results from the RC chain getting orientated along the force direction and the film punchout along the vertical direction. Generally, the more pre-stretching ratio of the film, the higher the orientation it will have. It is known that BNNS exhibits high TC along the (002) lattice plane, whereas the TC value is considerably declined along the (100) lattice plane. Hence, the ratio between the intensity of the (002) peak (*I*_002_) and the (100) peak (*I*_100_) is represented to characterize the anisotropic degree of BNNSs by XRD test in Figure S7, and the calculated *I*_002_*/I*_100_ values, which could reflect the in-plane-orientated degree of BNNS, are illustrated in Figure 3A. With the increase in the pre-stretching ratio from 100 to 400%, the value of *I*_002_*/I*_100_revealed a growing trend from 48 to 226, indicating that the orientation of the BNNS tend to be more prominent in the composite film after stretching. The cross-section SEM image of the dual-crosslinked RC/BNNS film after 400% stretching (Figure 3B) and its partial enlarged detail (Figure 3C) displayed that the BNNS lie horizontally along the in-plane direction of the composite film, which was visibly different from the disordered orientation of the BNNS in the dual-crosslinked RC/BNNS film without pre-stretching (Figure S8). Moreover, with the formation of the RC/BNNS anisotropic structure, the mechanical properties of the film were also affected to some extent. Figure 3D shows the stress–strain curves of the dual-crosslinked RC/BNNS films after different degrees of pre-stretching. As can be seen, in the increase of the stretching ratio, dual-crosslinked RC/BNNS film with 400% stretching ratio behaves with much enhanced tensile strength (107 MPa), but with depressed elongation, it can be explained as the decrease in slidable RC chain. By the way, exorbitant pre-stretching will spoil the mechanical properties of the film when it is over a ratio of 300% (Figures 3E,F). The exorbitant pre-stretching may cause the crack in the chemical crosslinking between the RC chain, so that it will weaken the modulus and toughness. In addition, the mechanical properties of the optimal RC/BNNS film were tested under 10,000 times of folding (Figure 3G), and the results show that the tensile strength declined slowly, while the toughness could be well-preserved, which is a direct evidence for its excellent flexibility. Moreover, this folded film could also withstand a 500-g weight that is almost 20,000 times of its own weight (Figure 3H), further proving its strong and flexible performance.

**Figure 4:**
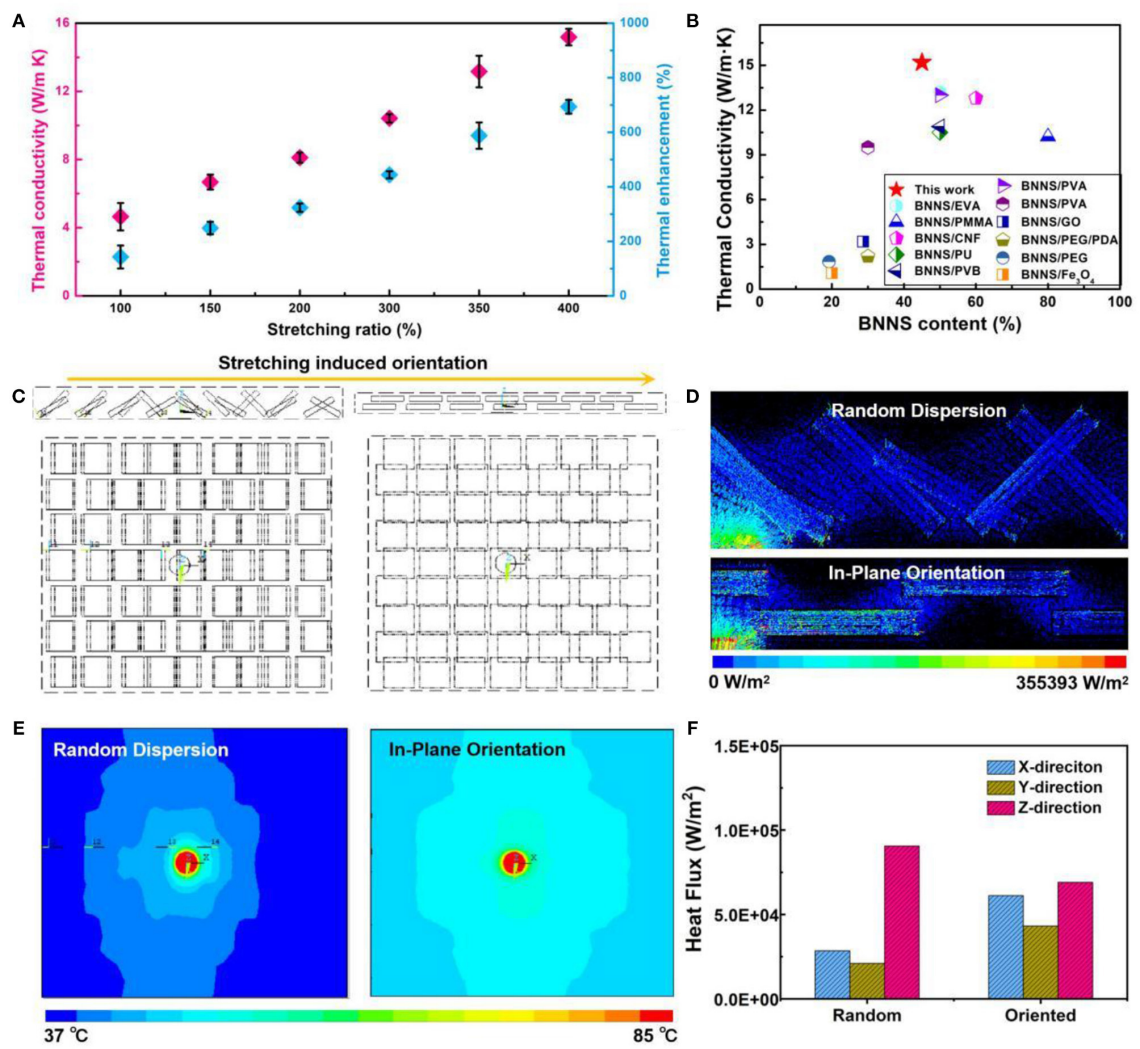
**(A)** Thermal conductivity (TC) and the corresponding thermal enhancements of the dual-crosslinked films after 100–400% stretching. **(B)** Comparison of TC as a function of BNNS content with previous reports. **(C)** The simulated arrangements of the BNNSs with random dispersion and in-plane orientation. Map of the **(D)** cross-section thermal distribution, **(E)** in-plane thermal distribution, and **(F)** the corresponding heat flux results of the BNNS-based models with random dispersion and in-plane orientation.

For the BNNS-based thermo-conductive material, the thermal conductivity is greatly affected by the directional arrangement of the BNNS in the matrix. As a result, with the increase in the pre-stretching ratio from 100 to 400%, the thermal conductivity of the dual-crosslinked RC/BNNS film was increased accordingly (Figure 4A). Note that the value of the film with 400% of pre-stretching reached to a high level of 15.2 W/m·K, which was almost 7.9 times higher than the pure RC film and 3.2 times higher than the film without pre-stretching. We also compare this composite film with most of the BNNS-based thermal conducting materials (Figure 4B); our dual-crosslinked RC/BNNS film after 400% stretching behaves with a much higher TC value at a low BNNS content (Lim et al., 2013; Ahn et al., 2014; Morishita and Okamoto, 2016; Yang et al., 2016a,b, 2018; Hong et al., 2017; Wu et al., 2017; Wang et al., 2020). Much of the researches (Wei et al., 2011; Yang et al., 2019) verified that the in-plane thermal conductivity of BNNSs is dozens of times higher than that in the out-plane direction. The thermal enhancement profits from the in-plane-arranged orientation of the BNNSs, which maximized the utilization of the in-plane thermal conductivity of BNNSs. An increasing number of orientated BNNSs, arranged along the in-plane of the film with the stretch ratio improved, led to the intensive thermal conductivity, which corresponds to the *I*_002_*/I*_100_values calculated from XRD (Figure 3A). Considering the relative mechanical properties of the composite film, the pre-stretching ratio of 400% was regarded as the optimized one in this study. Furthermore, to realize a visual analysis of the enhancement on the thermal conductivity, the finite element (FE) analysis was provided to simulate the heat fluxes and temperature distribution of the dual-crosslinked RC/BNNS films. The detailed simulation and parameters are listed in Figures S9, S10 and Table S1. Figure 4C illustrates two simulated arrangements of the BNNS with random dispersion and in-plane orientation in the RC matrix, and the hotspots were set in the middle of the upper surface. After heating, the radial heat flux transfer rate of the randomly dispersed model was obviously lower than that of the oriented model along the in-plane direction (x-direction and y-direction) as time went by Figure 4D. The heat diffusion of the oriented film was more obvious than that of the film with inefficient BNNS orientation, and the heat distribution was more uniform (Figure 4E). For intuitive represention of the heat flux along the different orientations, we count the heat flux distribution in the x-, y-, and z-directions (Figure 4F). The in-plane heat flux of the oriented model was superior to the random model, but with an inferior heat flux along the z-direction. Combined with Figure 4C, it is indicated that the in-plane heat flux seizes the principal position in the oriented model, which attributes to the in-plane-arranged orientation of the BNNSs.

**Figure 5:**
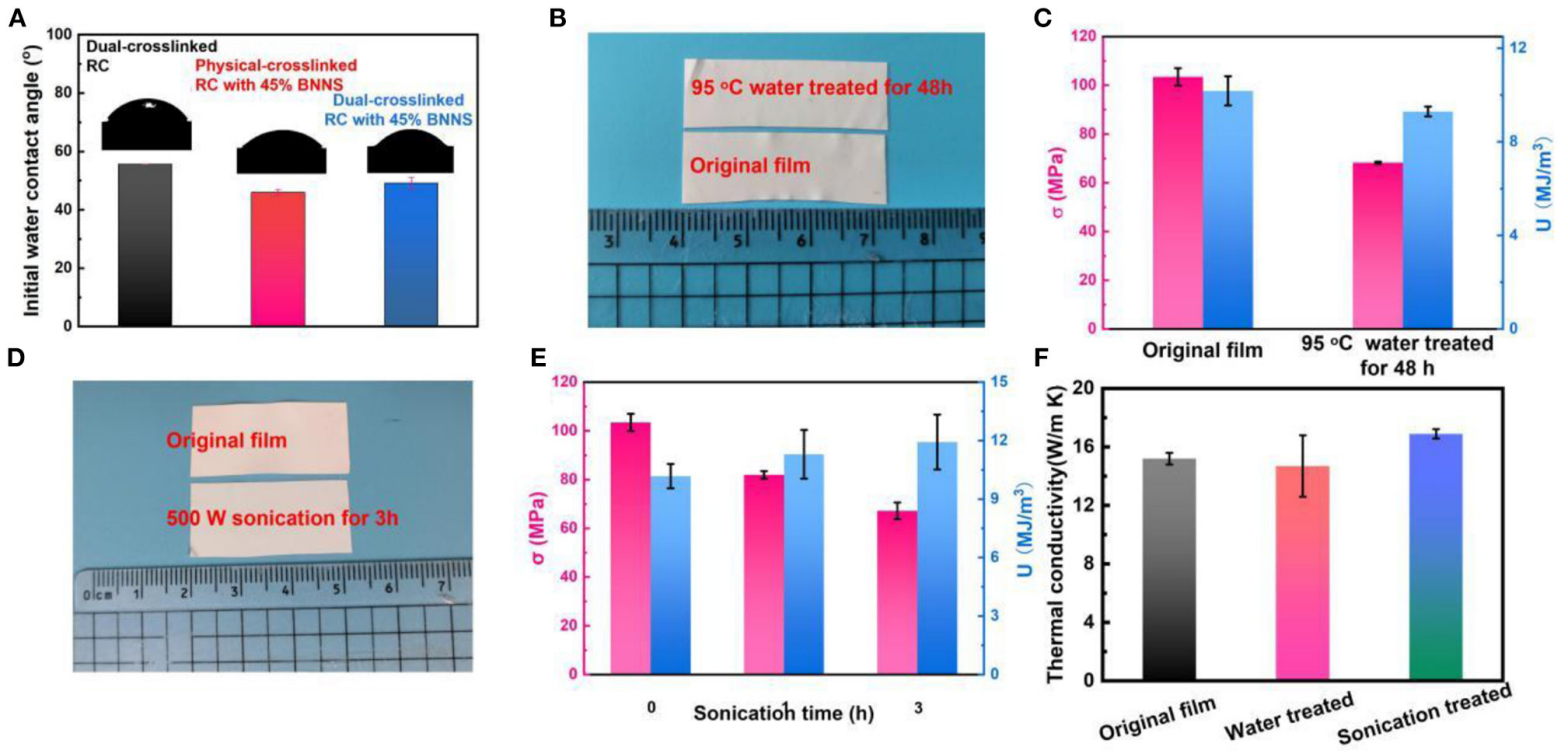
**(A)** Initial water contact angle results and photographs of water droplets on the surface of the samples. **(B)** Digital images and **(C)** mechanical properties of the original film and the film treated in 95°C water for 48 h. **(D)** Digital images and **(E)** mechanical properties of the original film and the film treated by 500 W sonication for 3 h. **(F)** Thermal conductivity of the RC/BNNS samples.

To investigate the water resistance of the thermo-conductive film, the surface hydrophilicity of the dual-crosslinked RC film, the physical crosslinked RC/BNNS film with 45% BNNS, and the dual-crosslinked RC/BNNS film with 45% BNNS were characterized first by water contact angle measurements (Figure 5A). The RC film exhibited a contact angle of 55.7°, while the two other samples exhibited slightly reduced contact angles, suggesting that the dual-crosslinked composite film maintained good hydrophilicity of cellulose. For the final optimized RC/BNNS film, the shape and mechanical properties were tested at 95°C water treated for 48 h (Figures 5B,C) and 500 W sonication for 3 h (Figures 5D,E), respectively. After being treated by the above two situations, the shape of the film was almost unchanged, and more importantly, the tensile strength and toughness were maintained at high levels. In addition, the thermal conductivity of the original, water treated, and sonication-treated films were also compared in Figure 5F, and the results further verify the qualified water resistance of the films.

## Conclusions

In summary, we report the fabrication of a strong, tough, and flexible RC/BNNS composite film by combining chemically and physically dual-crosslinking strategy. By stretching-induced orientation, the composite film possessed an anisotropic structure with highly ordered cellulose and BNNSs in the plane direction. As a result, the dual-crosslinked RC/BNNS composite film exhibited high tensile strength (103.4 MPa), high toughness (10.18 MJ/m^3^), and excellent thermal conductivity (15.2 W/m·K) with 400% pre-stretching. These properties were consistent with the results through finite element modeling calculation. Furthermore, the optimized RC/BNNS composite film presented excellent folding endurance of more than 10,000 times, and qualified stability under sonication and hydrothermal treatment. Such comprehensive properties greatly exceed that of conventional isotropic thermal conductive materials, which will broaden the practical applications in the portable and wearable electronic devices.

## Data Availability Statement

The original contributions presented in the study are included in the article/Supplementary Material, further inquiries can be directed to the corresponding authors.

## Author Contributions

The manuscript was written through contributions of all authors. All authors have given approval to the final version of the manuscript. All authors contributed equally.

## Conflict of Interest

The authors declare that the research was conducted in the absence of any commercial or financial relationships that could be construed as a potential conflict of interest.

## Supplementary Material

Supplementary Figure 1 Schematic diagram of the fabrication process of the anisotropic dual-crosslinked RC/BNNS films.

Supplementary Figure 2 TGA test of cellulose/BNNS supernatan.

Supplementary Figure 3 Viscosity test results of the pure cellulose solution, the cellulose/BNNS suspension, and the cellulose/BNNS/ECH suspension.

Supplementary Figure 4 FT-IR spectra of RC or RC/BNNS films.

Supplementary Figure 5 TGA and DSC curves of RC or RC/BNNS films.

Supplementary Figure 6 Cross-section SEM image and the partial enlarged detail of the dual-crosslinked RC/BNNS film with 60 wt% BNNS content after 300% pre-stretching.

Supplementary Figure 7 XRD results of the dual-crosslinked RC/BNNS films with different BNNS contents.

Supplementary Figure 8 Cross-section SEM image of the dual-crosslinked RC/BNNS film without pre-stretching.

Supplementary Figure 9 Heat flux in RC/BNNS films.

Supplementary Figure 10 FE analysis detail.

Supplementary Table 1 FE analysis detail.
