# Supplementary material for: A Dual-Crosslinked and Anisotropic Regenerated Cellulose/Boron Nitride Nanosheets Film With High Thermal Conductivity, Mechanical Strength, and Toughness
Source: Front Bioeng Biotechnol. 2020 Dec 18;8:602318. doi: 10.3389/fbioe.2020.602318 (PMC7775592; doi:10.3389/fbioe.2020.602318)
Supplement: Supplementary Figure 1 — Schematic diagram of the fabrication process of the anisotropic dual-crosslinked RC/BNNS films. [file Data_Sheet_1.DOCX]

Supporting Information

**A Dual-Crosslinked and Anisotropic Regenerated Cellulose/Boron Nitride Nanosheets Film with High Thermal Conductivity, Mechanical Strength and Toughness**

Xuran Xu ^2^ ^#^, Yichuan Su ^1 2 #^, Yongzheng Zhang ^1 2^, Shuaining Wu ^2^, Kai Wu ^1 2 3 *^, Qiang Fu ^1 *^

^1^ College of Polymer Science and Engineering, State Key Laboratory of Polymer Materials Engineering, Sichuan University, Chengdu 610065, China.

^2^ Key Laboratory for Soft Chemistry and Functional Materials of the Ministry of Education, School of Chemical Engineering, Nanjing University of Science and Technology, Nanjing 210094, China.

^3^ Key Laboratory of Advanced Technologies of Materials, Ministry of Education China, Southwest Jiaotong University, Chengdu, China

^#^ These two authors contributed equally to this work.

^*^ Corresponding author. E-mail: kaiwu@njust.edu.cn (Kai Wu).

^*^ Corresponding author. E-mail: qiangfu@scu.edu.cn (Qiang Fu).

**Calculation of Young’s modulus and toughness**

The Young’s mudulus (*E*) is defined as the ratio of positive stress to positive strain, and it is calculated as follow:

$\text{E}=\text{（F}\times L)/(A\times\Delta L)$ (1)

Where *F* refers to the stress, *L* refers to the initial length, *A* refers to the cross-section area, and *∆L* refers to the strain length.

Toughness (*U_r_*) is defined as the area between the stress-strain curve and the X-axis, and it is calculated as follow:

$U_{r}=\int\text{σdε}$ (2)

Where σ refers to the positive stress, and ε refers to the positive strain.

**Supportmentary Figures**


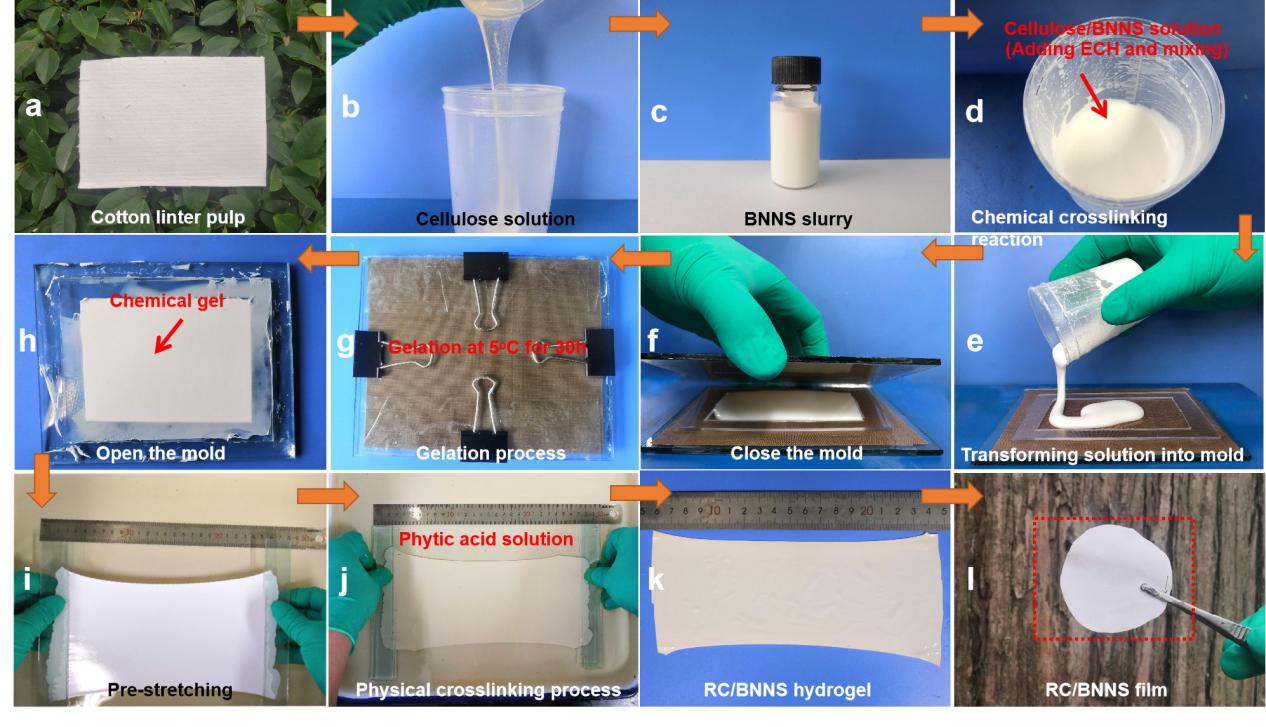


**Figure S1**. Schematic diagram of the fabrication process of the anisotropic and dual-crosslinked RC/BNNS films.


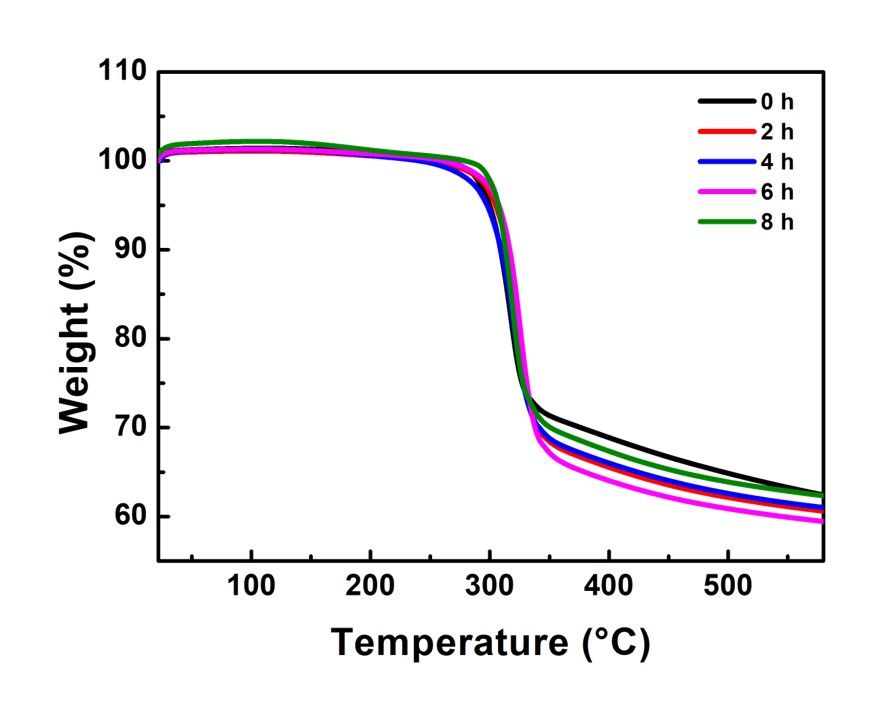


**Figure S2**. TGA curves of the cellulose/BNNS supernatant after different time.


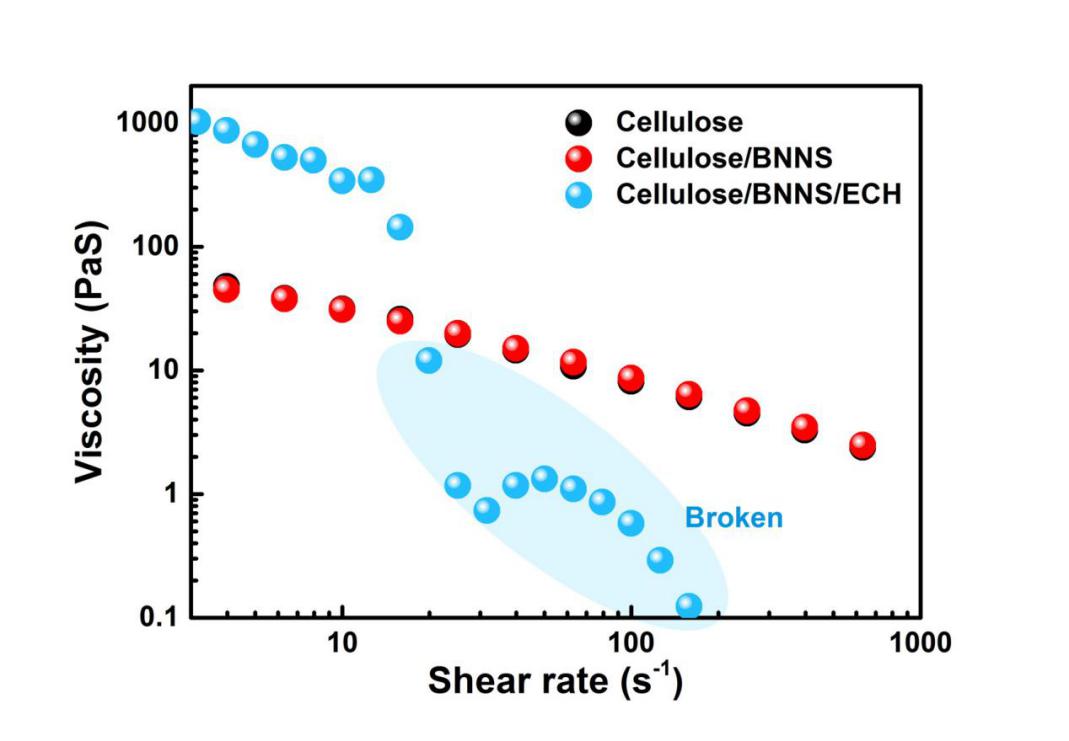


**Figure S3**. Rheology test results of the pure cellulose solution, the cellulose/BNNS suspension and the cellulose/BNNS/ECH suspension.


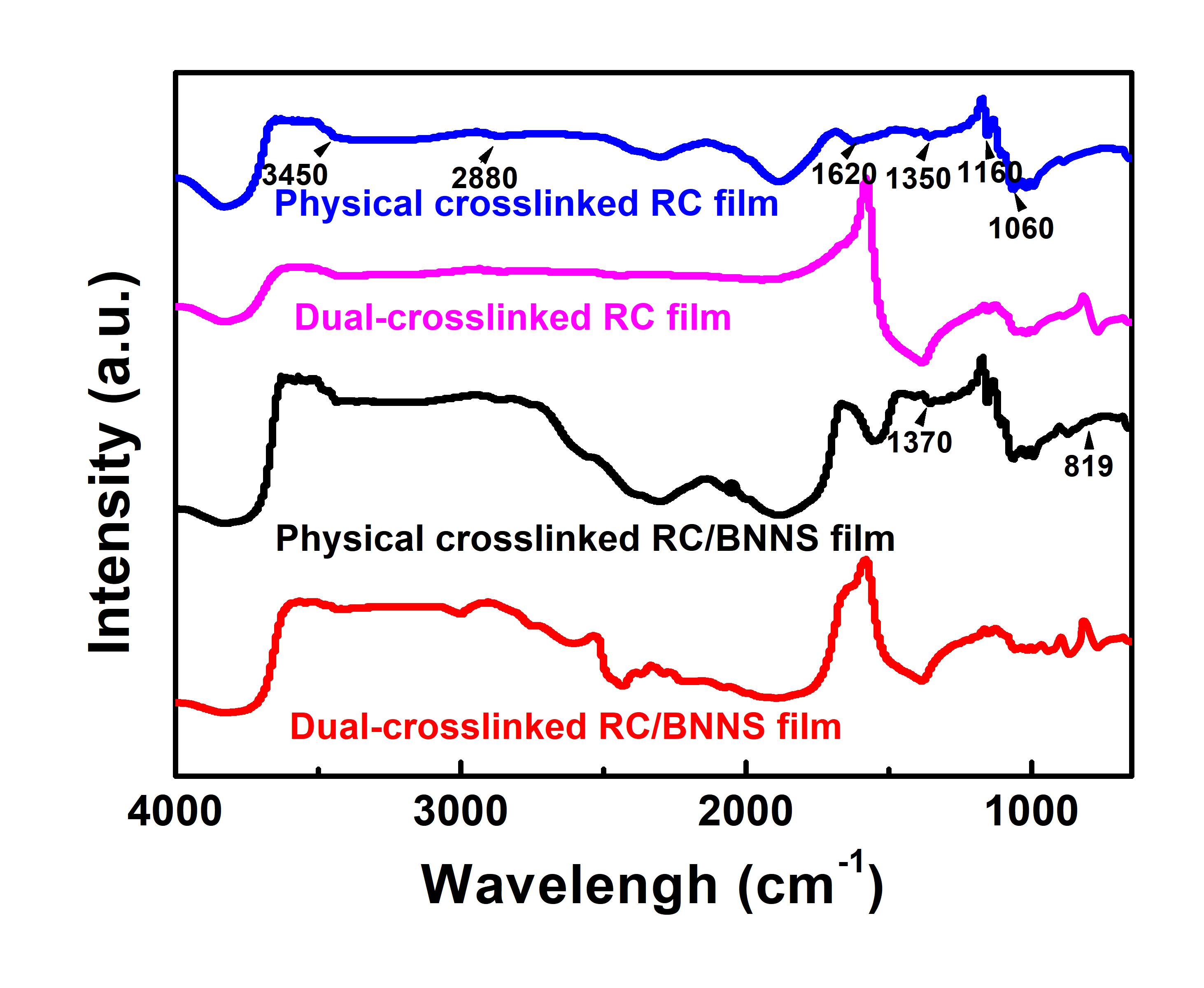


**Figure S4**. FT-IR spectra of physical crosslinked RC, dual-crosslinked RC, physical crosslinked RC/BNNS and dual-crosslinked RC/BNNS films.


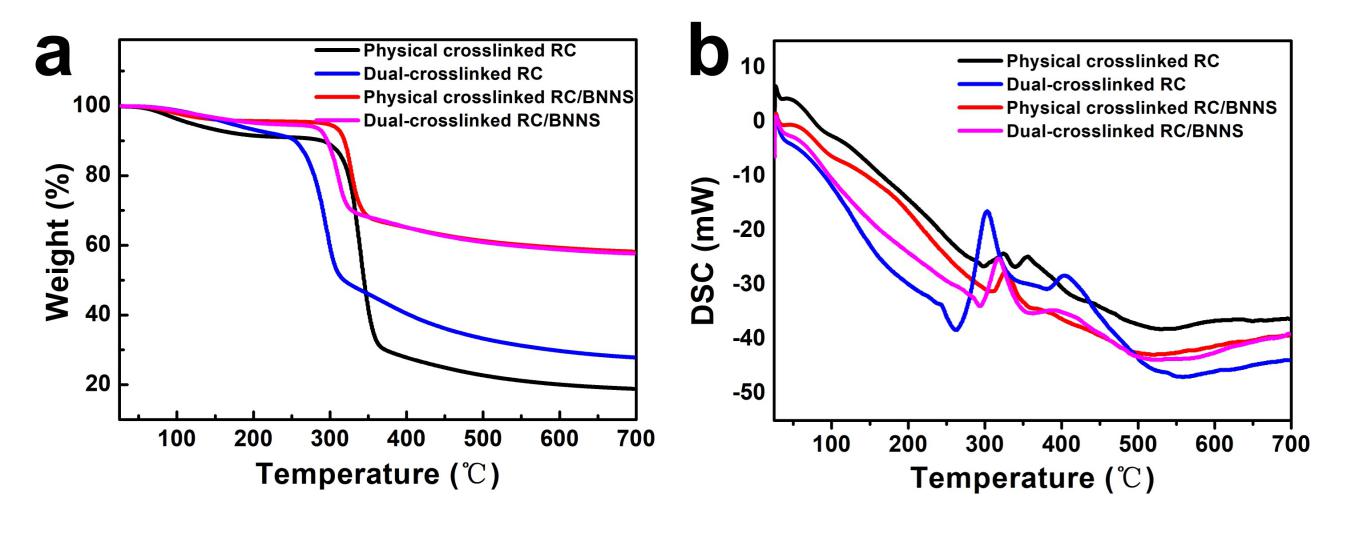


**Figure S5.** (a) TGA curves and (b) DSC curves of physical crosslinked RC, dual-crosslinked RC, physical crosslinked RC/BNNS and dual-crosslinked RC/BNNS.


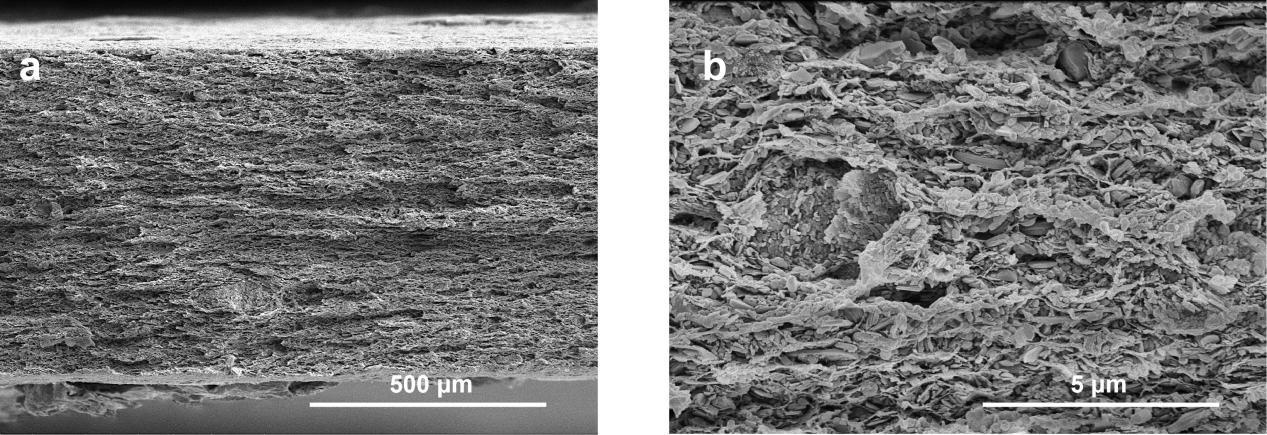


**Figure S6**. (a) Cross-section SEM image and (b) the partial enlarged detail of the dual-crosslinked RC/BNNS film with 60 wt% BNNS content after 400 % pre-stretching.


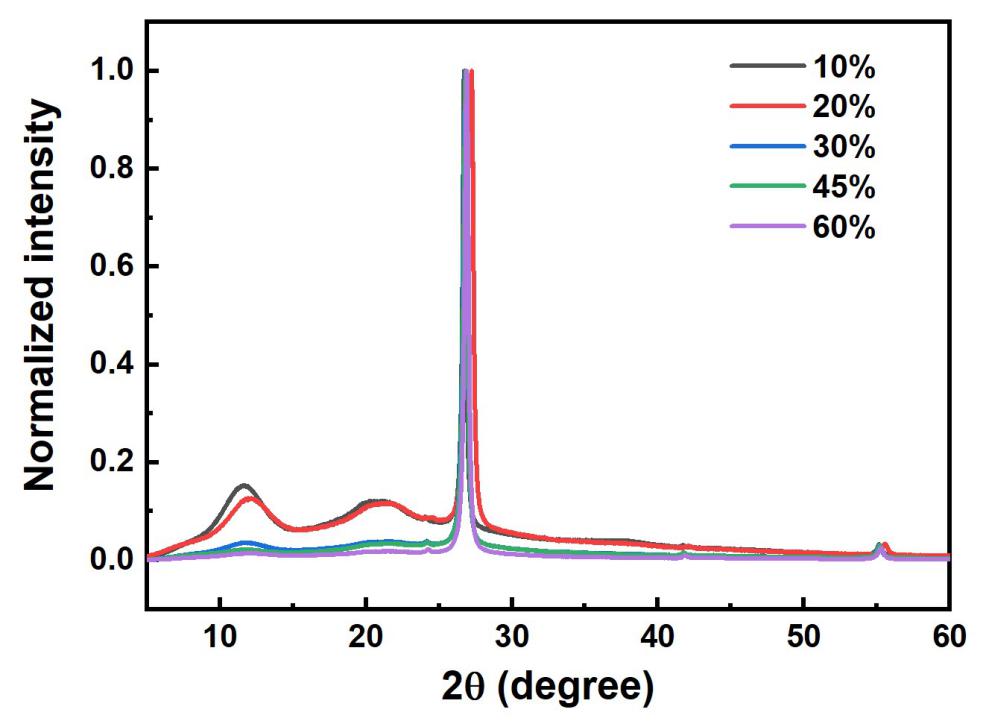


**Figure S7**. XRD results of the dual-crosslinked RC/BNNS films with different BNNS contents.


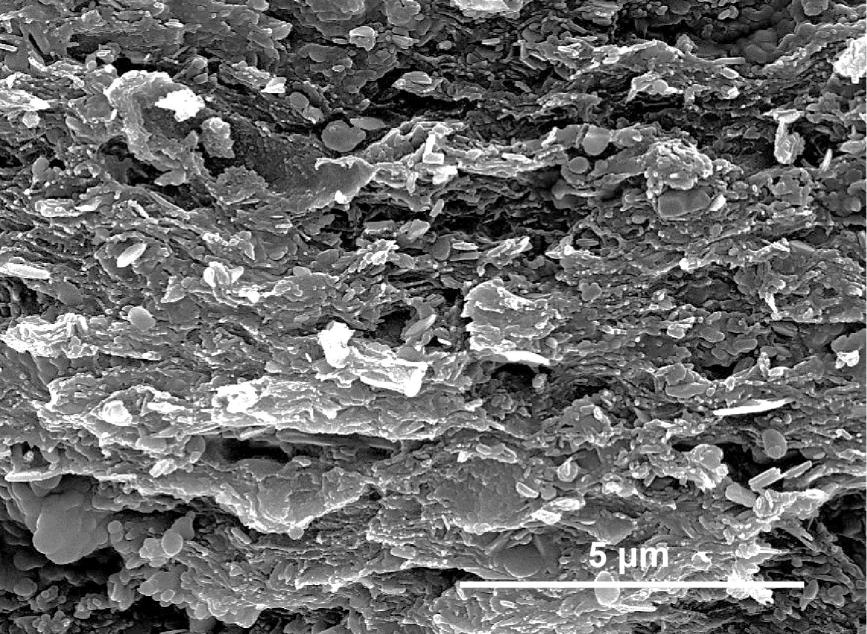


**Figure S8**. Cross-section SEM image of the dual-crosslinked RC/BNNS film without pre-stretching.


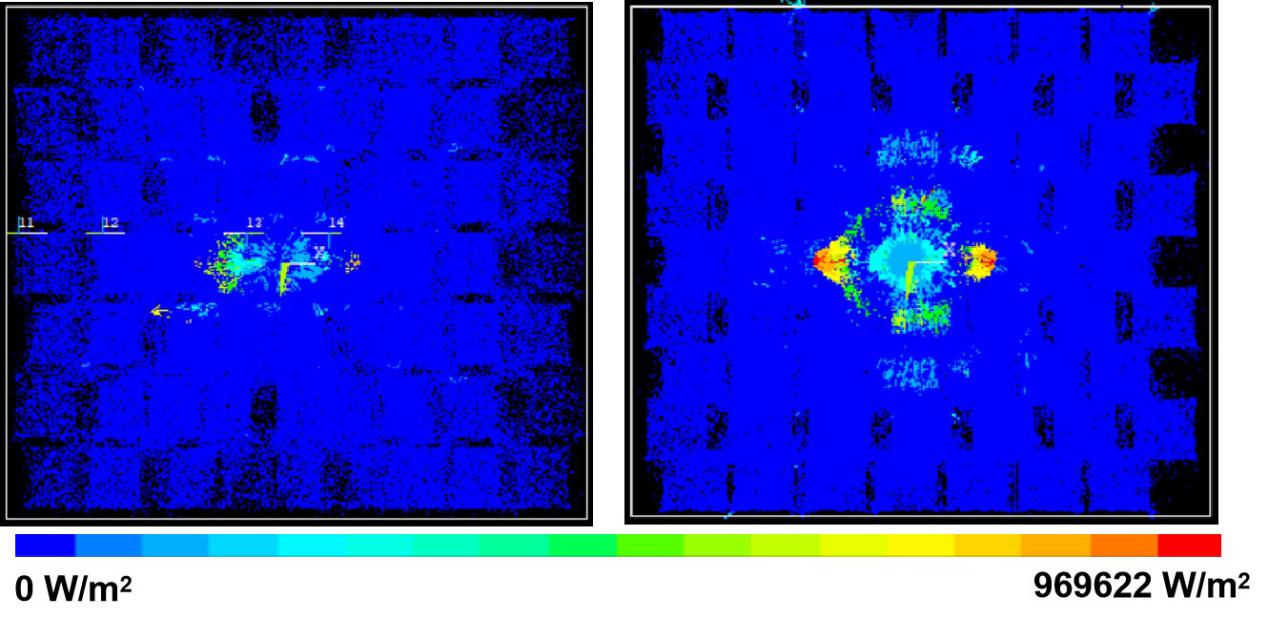


**Figure S9**. Heat flux of the dual-crosslinked RC/BNNS film with randomly distributed BNNS (left) and in-plane orientation of BNNS (right).

**FE analysis**


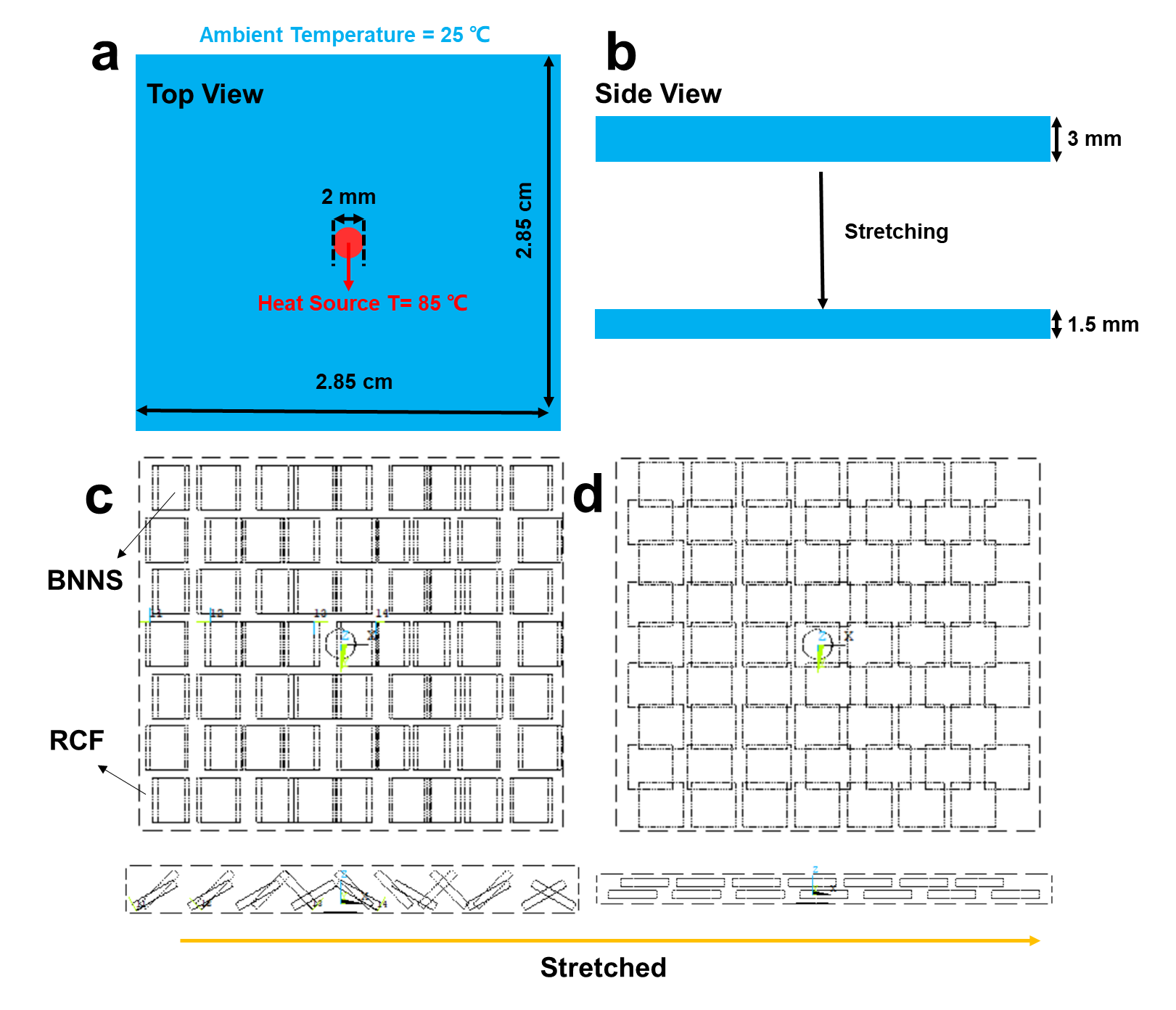


**Figure S10**. (a) The top view and (b) side view of the simulative model and boundary conditions for finite element analysis. The model of BNNS within (c) random RC/BNNS and (d) oriented RC/BNNS for finite element analysis.

**Table S1**. Parameters for the finite element simulation analysis.

| **Parameters** | **RC** | **BNNS** | |
| --- | --- | --- | --- |
| Thermal conductivity (W/m K) | 0.4 | In-Plane | Through-Plane |
|  |  | 600 | 10 |
| Specific heat capacity (J/(g ℃)) | 1160 | 1050 | |
| Density (kg/m^3^) | 1470 | 2290 | |
